# Quinoxaline Moiety: A Potential Scaffold against *Mycobacterium tuberculosis*

**DOI:** 10.3390/molecules26164742

**Published:** 2021-08-05

**Authors:** Marc Montana, Vincent Montero, Omar Khoumeri, Patrice Vanelle

**Affiliations:** 1 Aix Marseille Univ, CNRS, ICR, Equipe Pharmaco-Chimie Radicalaire, Faculté de Pharmacie, 13005 Marseille, France; marc.montana@univ-amu.fr (M.M.); vincent.montero@univ-amu.fr (V.M.); omar.khoumeri@univ-amu.fr (O.K.); 2 Assistance Publique-Hôpitaux de Marseille (AP-HM), Oncopharma, 13015 Marseille, France; 3 Assistance Publique-Hôpitaux de Marseille (AP-HM), Service Central de la Qualité et de l’Information Pharmaceutiques (SCQIP), 13005 Marseille, France

**Keywords:** quinoxaline, *Mycobacterium*, tuberculosis, SAR, biological applications, chemistry

## Abstract

**Background**. The past decades have seen numerous efforts to develop new antitubercular agents. Currently, the available regimens are lengthy, only partially effective, and associated with high rates of adverse events. The challenge is therefore to develop new agents with faster and more efficient action. The versatile quinoxaline ring possesses a broad spectrum of pharmacological activities, ensuring considerable attention to it in the field of medicinal chemistry. **Objectives**. In continuation of our program on the pharmacological activity of quinoxaline derivatives, this review focuses on potential antimycobacterial activity of recent quinoxaline derivatives and discusses their structure—activity relationship for designing new analogs with improved activity. **Methods**. The review compiles recent studies published between January 2011 and April 2021. **Results**. The final total of 23 studies were examined. Conclusions. Data from studies of quinoxaline and quinoxaline 1,4-di-*N*-oxide derivatives highlight that specific derivatives show encouraging perspectives in the treatment of *Mycobacterium tuberculosis* and the recent growing interest for these scaffolds. These interesting results warrant further investigation, which may allow identification of novel antitubercular candidates based on this scaffold.

## 1. Introduction

In 2020, the World Health Organization estimated that about 10 million people (range: 8.9–11.0 million) contracted tuberculosis (TB) in 2019, which was responsible for 1.4 million deaths [1]. Treatment of drug-susceptible active tuberculosis consists of a standard 6-month regimen of four antimicrobials (usually isoniazid, rifampin, pyrazinamide, and ethambutol) [2,3]. However, these regimens are lengthy, only partially effective, and associated with high rates of adverse events. The challenge is therefore to develop new agents with faster and more efficient action.

Nitrogen-containing heterocycles are of particular interest for the development of new drugs or novel potential lead molecules [4,5,6,7,8]. Quinoxaline, formed by the fusion of two aromatic rings, benzene and pyrazine, is one of the heterocycles receiving the most attention.

**Figure 1:**
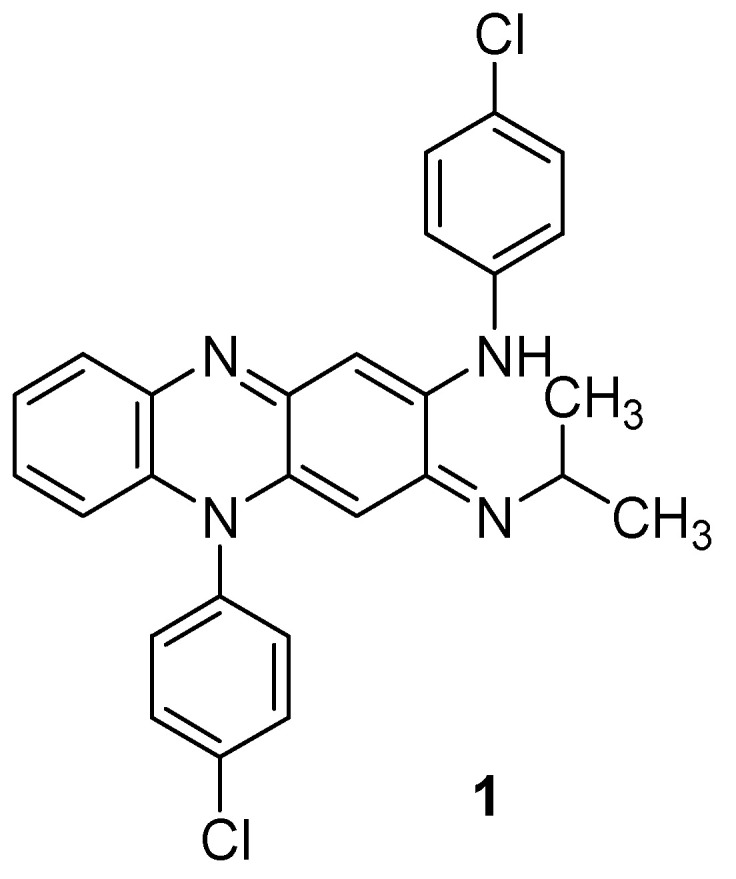
Chemical structure of clofazimine.

The versatile quinoxaline ring possesses a broad spectrum of pharmacological activities (antiviral [9], anticancer [10], antileishmanial [11]), ensuring considerable attention to it in the field of medicinal chemistry [12]. In addition, as quinoxaline is also a part of well-known wide-spectrum antibiotics echinomycin, levomycin, and actinoleutin, quinoxaline derivatives are expected to have antimycobacterial activity. One of them, clofazimine, initially known as B663, is currently under intensive clinical investigation for the treatment of drug-resistant TB to assess the compound’s treatment outcomes with multidrug-resistant and extensively drug-resistant TB (Figure 1).

Some quinoxaline-1,4-di-*N*-oxide derivatives have also shown excellent antimicrobial activities against *Mycobacterium tuberculosis* [13,14,15], indicating the great interest of these types of structure for the development of new structural classes of anti-TB drugs. The oxidation of nitrogen in a quinoxaline ring has a pronounced effect on antimycobacterial activity [16]. In addition, quinoxaline 1,4-di-*N*-oxides are species that suffer a bioreductive process under the hypoxic conditions [17] found in tuberculous granulomas, where nonreplicating persistent forms of *Mycobacterium tuberculosis* bacilli can survive, leading to the need for long treatments and the risk of treatment resistance [13]. In continuation of our program on the pharmacological activity of quinoxaline derivatives, we decided to focus on the development of new quinoxalines and quinoxaline 1,4-di-*N*-oxide derivatives as antitubercular drugs. This review compiles studies published between 2011 and 2021 and discusses the potential antimycobacterial activity of recent quinoxaline derivatives. 

## 2. Methods

### 2.1. Data Sources and Searches

The research was conducted using three databases: MEDLINE/PubMed, Web of Science, and Science Direct Elsevier (Table 1).

### 2.2. Study Selection

The review was performed by two independent reviewers as described by PRISMA [18]. Eligibility criteria were predetermined by the authors (Table 2). 

In the first step, duplicates were eliminated. Then, articles’ titles and abstracts were evaluated according to the inclusion criteria. The authors read each selected full text and eliminated articles fitting the exclusion criteria. During this stage, the references of the relevant articles were examined to identify additional studies not retrieved in computerized databases.

## 3. Results

The database search identified 148 records; 27 repeated files were discarded, leaving 121 articles.

After the evaluation phase (title/abstract) and full-text reading, 98 records were excluded. The final total of 23 studies were included in this review. No other paper was added from the reference lists of the identified studies.

During the 2011–2015 and the 2016–2021 periods, six articles (two concerning 1,4-di-*N*-oxide-quinoxaline derivatives and four concerning quinoxaline derivatives) and 17 articles (six concerning 1,4-di-*N*-oxide-quinoxaline derivatives and 11 concerning quinoxaline derivatives) were published, respectively.

## 4. Discussion

### 4.1. Quinoxaline Derivatives as Antimycobacterial Agents

A wide variety of methods for the synthesis of functionalized quinoxalines have already been reported in the literature. In general, quinoxaline derivatives are obtained either by condensation of *o*-phenylenediamine and its derivatives with a dicarbonyl compound under conventional conditions or reaction of 1,2-diaza-1,3-butadienes with 1,2-diamines [19,20,21,22]. Eco-friendly approaches using recyclable catalysts [23,24], oxidative cyclisation of 1,2-diamines and phenacyl bromides [25], microwave-assisted synthesis [26,27], reactions in aqueous medium [28], or one-pot synthesis [29] have also been described.

#### 4.1.1. Tricyclic Quinoxaline Derivatives

**Figure 2:**
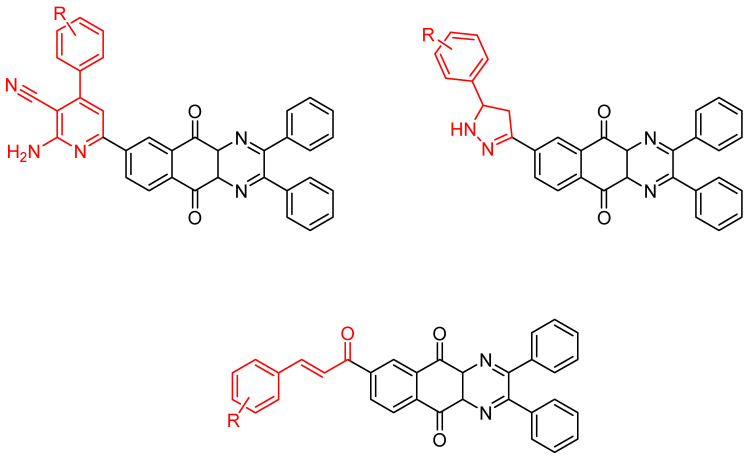
Chemical structures of new series benzo[*g*]quinoxaline-5,10-dione derivatives.

Between 2017 and 2020, three series of original benzo[*g*]quinoxaline-5,10-dione derivatives were synthesized using a multistep synthetic route from 2,3-dihydroxynaphtalene and evaluated as potential antimycobacterial agents [30,31,32] (Figure 2).

Thirteen novel compounds belonging to the pyridine series, 11 new pyrazoline derivatives, and 11 7-[3-(substituted) phenylprop-2-enoyl]quinoxaline derivatives were obtained; they displayed moderate antimycobacterial activity [30,31,32] (Table 3).

**Figure 3:**
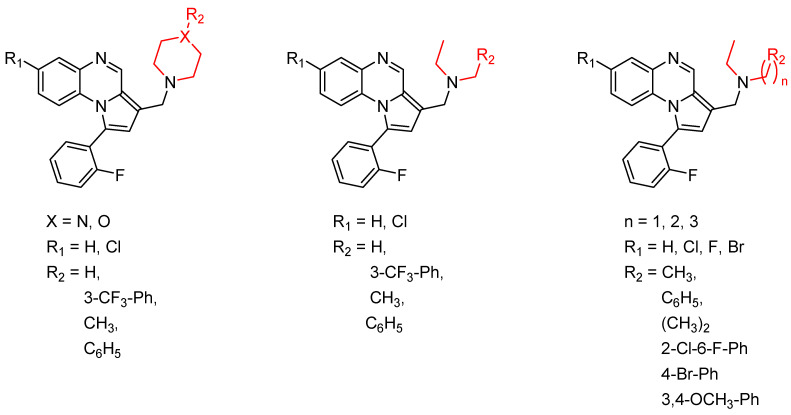
Chemical structures of new series of pyrrolo[1,2-*a*]quinoxaline derivatives.

Pyrroloquinoxaline moiety represents a very attractive scaffold in medicinal chemistry for its biological anti-infectious properties, including antimycobacterial activity [33]. This, pyrrolo[1,2-*a*]quinoxaline was chosen as scaffold and led to the synthesis of 23 new pyrrolo[1,2-*a*]quinoxaline derivatives (Figure 3) that displayed very encouraging antimycobacterial activity [34].

Ten compounds showed both interesting antimycobacterial activity and moderate cytotoxicity. Antimycobacterial activity was observed to depend on varying chemical characteristics, like the replacement of tertiary nitrogen by an NH group, leading to compounds **7**–**10** with improved solubility and the lowest minimal inhibitory concentration (MIC) value (5 µg/mL) (Table 4).

**Figure 4:**
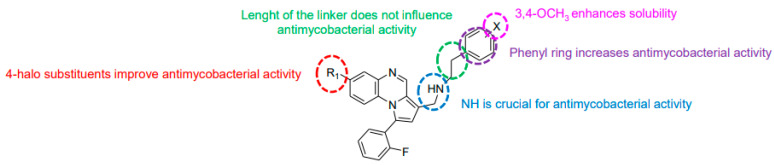
Structure–activity relationship of novel quinoxaline derivatives with antimycobacterial activity.

Modification of the linker between R_2_ and pyrrolo[1,2-*a*]quinoxaline moiety led to a minor variation in antibacterial activity, although substitutions at the R_2_ position with an aromatic group led to the most active compounds (Figure 4). Derivative *N*-((7-chloro-1-(2-fluorophenyl)pyrrolo[1,2-*a*]quinoxalin-3-yl)methyl)-2-(3,4-dimethoxyphenyl)ethanamine (**9**) was identified as offering the best oral bioavailability. Determination of enzyme inhibition and molecular docking studies revealed that these original derivatives could act by targeting the classic anti-TB drug target InhA by forming H bonds as well as Pi–Pi interactions.

**Figure 5:**
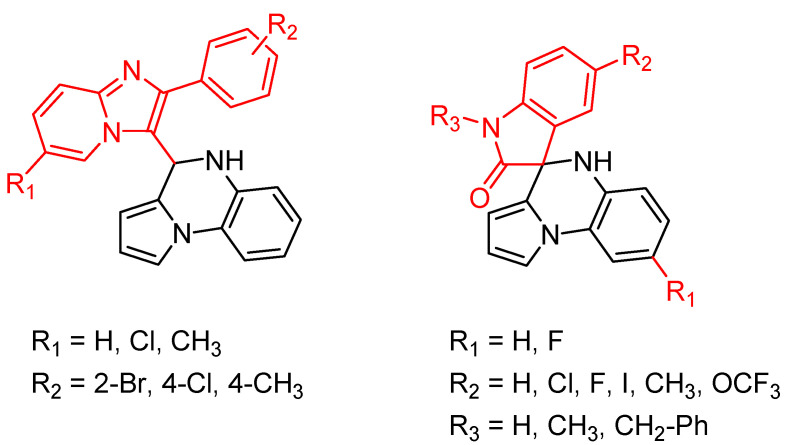
General structure of novel 4,5-dihydropyrrolo[1,2-*a*]quinoxalines and pyrrolo[1,2-*a*]quinoxaline-2-ones synthesized.

In addition, several novel 4,5-dihydropyrrolo[1,2-*a*]quinoxalines and pyrrolo[1,2-*a*]quinoxaline-2-ones were synthesized from the reaction between 2-(1*H*-pyrrol-1-yl)anilines and imidazo[1,2-*a*]pyridine-3-carbaldehyde or isatin and are of current interest as antimycobacterial agents [35] (Figure 5).

Five 4-substitued 4,5-dihydropyrrolo[1,2-*a*]quinoxalines and only one pyrrolo[1,2-*a*]quinoxaline-2-one derivative displayed potent antimycobacterial activity, with MIC ranging from 6.25 to 12.5 µg/mL. However, no clear structure–activity relationship could be demonstrated from the small number of synthesized compounds [35].

#### 4.1.2. Hybrids and Conjugates

Many classes of organic compounds have previously been evaluated for their anti-mycobacterial activity. A rational design strategy based on combining the biological properties of different bioactive structures into a single compound could lead to new compounds with increased activity, combined modes of action, or improved tolerance profiles compared to parent structures.

**Figure 6:**
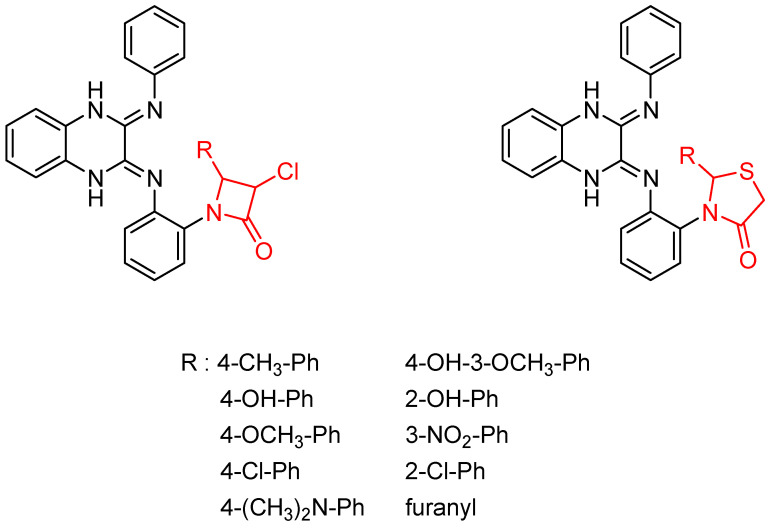
General structure of original quinoxaline derivatives containing azetidinone and thiazolidinone moieties.

Then, the quinoxaline ring fused with azetidinone and thiazolidinone, two structural scaffolds possessing antitubercular activity [36,37,38], showed in vitro activity against *Mycobacterium tuberculosis*. Quinoxaline derivatives with 2-chloro, dimethylamino and nitro substitution showed comparable activity to that of isoniazid (MIC = 0.67–0.97 µg/mL versus 0.46 µg/mL) [39] (Figure 6).

**Figure 7:**
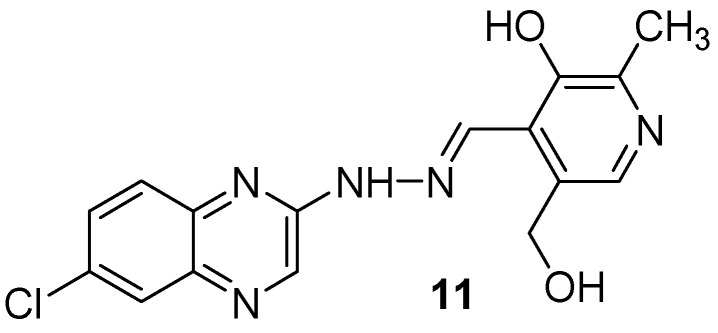
Lead compound of hydrazones and *N*-acylhydrazones containing vitamin B6 and different heteroaromatic nuclei.

As vitamin B6 is an important cofactor in a large number of important enzymic reactions and is also used in bioinorganic chemistry as a ligand, a new series of 13 quinoxalines bearing the pyridoxal moiety was synthesized and tested against *Mycobacterium tuberculosis* (Figure 7). In a hydrazone series, the 7-chloroquinoxaline derivative (**11**) showed the best activity, with the MIC of 72.72 µM. The number of nitrogen and chlorine atoms in the radical moiety plays an important role in antimycobacterial activity [40].

**Figure 8:**
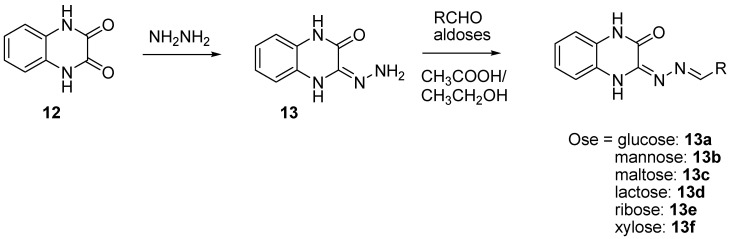
Schematic route for the synthesis of sugar conjugates of quinoxaline.

In order to prevent excessive toxicity due to high lipophilicity and considering the biosafety profile of sugar conjugates [41,42,43], some new sugar conjugates of quinoxaline were synthesized and evaluated for their antitubercular activity. Of a series of six sugar conjugates of quinoxaline compounds, all the synthesized derivatives demonstrated interesting antimycobacterial activity, with the MIC ranging from 0.65 to 9.6 µM [43] (Figure 8).

The ribose conjugate (**13e**) exhibited the best antimycobacterial activity, with the MIC = 0.65 µM. All the sugar conjugates were more active than the initial quinoxaline-2,3-(1*H*,4*H*)-dione and the intermediate 3-hydrazono-3,4-dihydroquinoxalin-2(1*H*)-one at the MIC > 59.2 µM and the MIC = 27.2 µM, respectively. The structure–activity relationship showed that monosaccharides had better activity than disaccharides and that the aldopentoses were more potent than the aldohexoses. As in silico docking analysis revealed that these compounds could interact with DNA gyrase, it was concluded that the stereoisomerism of sugar may influence antimycobacterial activity; this was supported by the fact that the ribose derivative was seven times more potent than the xylose derivative.

Chalcones are compounds with a wide range of biological activities, including antitubercular activity [44,45]. Of a series of 14 original quinoxalinyl chalcones, two derivatives demonstrated equivalent activity to pyrazinamide and ciprofloxacin taken as reference with the MIC = 3.12 µg/mL. The structure–activity relationship showed that the presence of a hydroxyl substituent at position 3 on the phenyl ring increases antimycobacterial activity. Replacing phenyl with naphthyl resulted in reduced antimycobacterial activity, indicating that the hydrophobicity/lipophilicity balance plays an important role in these derivatives’ activity [46] (Table 5).

The efficacy of quinoxaline-derived chalcones has recently been confirmed by the preclinical evaluation of a new series of derivatives. Of the synthesized compounds, six molecules inhibited *Mycobacterium tuberculosis* growth, with the MIC ranging from 3.13 to 12.5 µg/mL [47]. Further investigations on the lead compound (**20**) also demonstrated that this derivative exhibited synergistic effect with moxifloxacin and did not cause mutagenicity or genotoxicity (Table 6).

**Figure 9:**
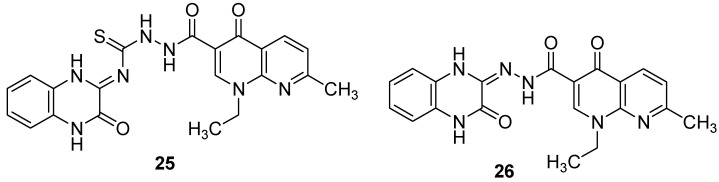
Chemical structures of lead compounds in nalidixic acid series.

In addition, the modification of nalidixic acid, a representative of the quinolone and fluoroquinolone antibiotics which are an essential component of treatment strategies for drug-resistant TB [1], on its –COOH group led to the formation of original quinoxaline conjugates which showed encouraging antimycobacterial activity. Among the synthesized derivatives, quinoxalines with azide as side chain (**25** and **26**) exhibited the two best activities, with the percentage of inhibition of *Mycobacterium tuberculosis* at 6.25 µg/mL of 93% and 91%, respectively, compared to 100% for ciprofloxacin taken as reference [48] (Figure 9).

**Figure 10:**
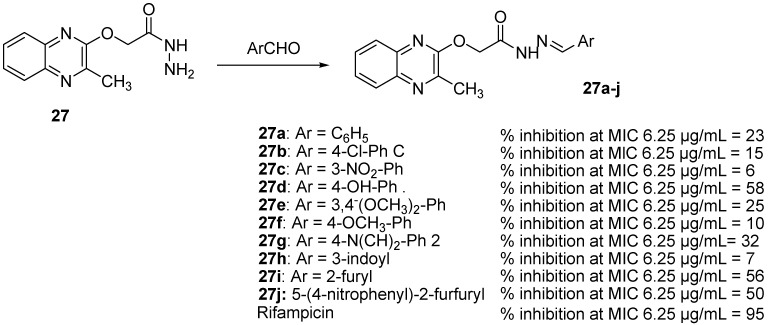
Percentage of inhibition of quinoxaline-incorporated Schiff bases against *Mycobacterium tuberculosis*.

As Schiff bases possess antimicrobial and antitubercular properties [49], synthesis of some quinoxaline-incorporated Schiff bases was performed. Among an original series of ten different Schiff bases resulting from the reaction between 2-((3-methylquinoxalin-2-yl)oxy)acetohydrazide (**31**) and various heterocyclic/aromatic aldehydes, five compounds exhibited potent antitubercular activity [49] (Figure 10).

Finally, spiroheterocyclic structures are well known for their antimycobacterial properties [50,51]. Spiropyrrolidine tethered indenoquinoxaline heterocyclic hybrids were synthesized and evaluated against *Mycobacterium tuberculosis*. Of the 11 synthesized compounds, bearing *m*-nitro, *p*-bromo and *o*-chloro substituents on the aryl ring showed interesting antimycobacterial activity, with MIC values ranging from 1.56 to 6.25 µg/mL [52] (Table 7).

#### 4.1.3. Other Structures

Several derivatives of 2-substituted quinoxalines have also shown interesting antitubercular activities. Thus, quinoxaline alkynyl derivatives demonstrated antimycobacterial activity. Of the 19 original quinoxalines bearing diverse substituents on the alkynyl group synthesized from quinoxaline-2-ol derivatives, seven compounds had MIC_90_ < 10 µM and five compounds had MIC_90_ ranging from 10 to 20 µM [53] (Table 8).

The presence of an electron-withdrawing group at the C6 position of the quinoxaline moiety plays an important role in antimycobacterial activity: four of the most active compounds are derivatives bearing NO_2_ at this position with MIC_90_ < 3 µM.

**Figure 11:**
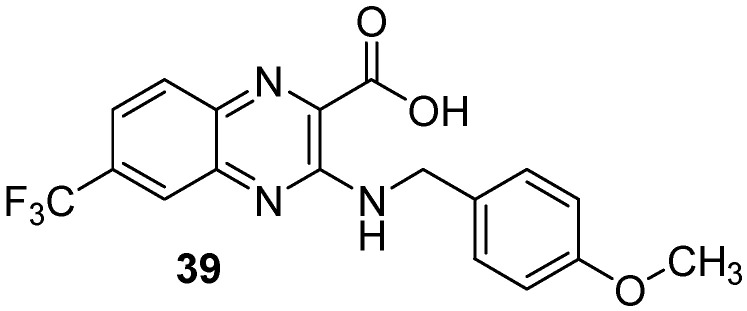
Structure of the lead compound 3-((4-methoxybenzyl)amino)-6-(trifluoromethyl)quinoxaline-2-carboxylic acid, a new DprE1 inhibitor.

Moreover, screening of a library of compounds identified three original 2-carboxyquinoxaline compounds showing activity against *Mycobacterium tuberculosis* with MIC_99_ values ranging from 3.1 to 12.5 µM [54]. Among them, the lead compound (**39**) which exhibited a MIC_99_ of 3.1 µM was shown to noncovalently and noncompetitively inhibit DprE1, an enzyme essential for bacterial wall synthesis by forming hydrophobic interactions and hydrogen bonds (Figure 11).

### 4.2. Quinoxaline-1,4-di-N-oxide Derivatives Active against Mycobacterium tuberculosis

Quinoxaline-1,4-di-*N*-oxide derivatives are a class of compounds with a variety of biological properties, including antitumor, anti-inflammatory, and anti-infectious activity [2]. The classic method of quinoxaline-1,4-di-*N*-oxide preparation uses benzofuroxane *N*-oxide as the reagent [55].

#### 4.2.1. Hybrids and Conjugates

**Figure 12:**
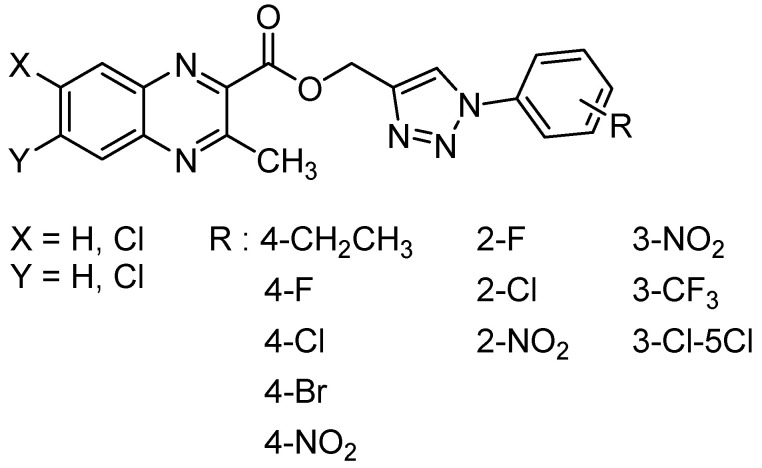
General structure of 1,2,3-triazole analogs of quinoxaline-1,4-di-*N*-oxide.

1,2,3-triazole and their derivatives are known for their various biological activities including antifungal and antibacterial activity [56]. In order to study the influence of the substitution at the C2 position in quinoxaline-1,4-di-*N*-oxide series, thirty-one 1,2,3-triazole analogs of quinoxaline-1,4-di-*N*-oxide were synthesized and evaluated against *Mycobacterium tuberculosis,* yielding 16 compounds with the MIC ranging from 12.5 to 25 µg/mL [56] (Figure 12).

More specifically, in 2-(((1-(substituted phenyl)-1*H*-1,2,3-triazol-4-yl)methoxy)carbonyl)-3-methylquinoxaline-1,4-dioxide series, only 2-chloro and 2- or 3-nitro compounds exhibited high activity. In 3-(((1-(substituted phenyl)-1*H*-1,2,3-triazol-4-yl)methoxy)carbonyl)-6-chloro-2-methylquinoxaline-1,4-dioxide series, unsubstituted, *ortho*-, and *para*-substituted derivatives exhibited high activity. The most active compound of the three series, 6-chloro-2-methyl-3-(((1-phenyl-1*H*-1,2,3-triazol-4-yl)methoxy)carbonyl)quinoxaline-1,4-dioxide, exhibited good antimycobacterial activity, with the MIC of 12.5 μg/mL against the tested strains versus 3.1 µg/mL for reference drug isoniazid. In the 2-(((1-(substituted phenyl)-1*H*-1,2,3-triazol-4-yl)methoxy)carbonyl)-6,7-dichloro-3-methylquinoxaline-1,4-dioxide series, only *para*-substituted halogen compounds exhibited activity. These structure–activity relationships also demonstrated that the introduction of an electron-withdrawing group resulted in less active compounds.

In addition, hybridization of quinoxaline 1,4-di-*N*-oxide with chalcone, fluoroquinolone, and thiazolidinone scaffolds was investigated [57,58]. Of the 10 original chalcones newly synthesized by Claisen–Schmidt condensation, only one displayed potency, with the MIC = 3.1 µM compared to 6.2 µM, 0.3 µM, and 0.04 µM, respectively, for ethambutol, isoniazid, and rifampicin taken as reference. In this same study, four of the five most potent derivatives were fluoroquinolone analogs, with MIC values ranging from 1.6 to 3.1 µM (Table 9). The structure–activity relationship showed that the presence of an electron-withdrawing substituent like a halogen atom at position C6 or C7 of the quinoxaline moiety enhances the activity. In addition, the presence of CF_3_ at position C3 promotes antimycobacterial activity, while the nature of the lateral side chain substituent at the C2 position of the quinoxaline group seems to have less influence on biological activity [57].

In thiazolidinone series, out of 26 novel derivatives, four compounds displayed potent antimycobacterial activity, four compounds—moderate activity [58] (Table 10). The presence of an electron-withdrawing group at the *para* position of the phenyl group is essential for higher activity and the presence of a halogen atom at position C7 of the quinoxaline nucleus increases antimycobacterial activity. Replacement of halogen atoms with a methyl or methoxy resulted in decreased antimycobacterial activity.

**Figure 13:**
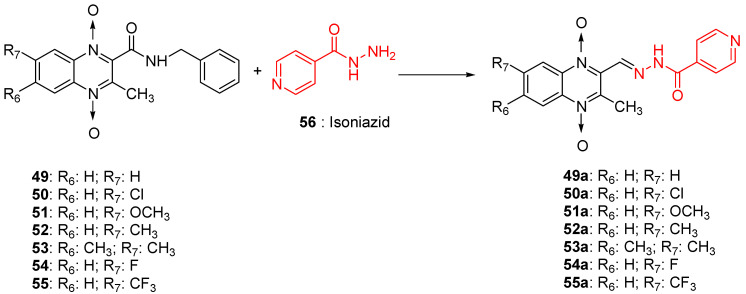
Design of 1,4-di-*N*-oxide quinoxaline-2-ylmethylene isonicotinic acid hydrazide derivatives.

As many isonicotinic acid hydrazide derivatives such as isoniazid demonstrated interesting anti-TB activities, an original series of hybrids resulting from the fusion between quinoxaline 1,4-di-*N*-oxide and isoniazid was synthetized; it showed high activity against *Mycobacterium tuberculosis*, with IC_90_ ranging from <1.16 to 50.60 µM versus 0.21 µM for isoniazid taken as reference (Figure 13). As isonicotinic hydrazide derivatives can be considered as a prodrug, these new hybrids could act according to the original mechanism of action where the hydrolysis of the compounds would lead to the formation of isoniazid which would act according to its own mechanism of action and of quinoxaline derivatives which would act synergistically with isoniazid. Position 7 unsubstituted or substituted by an electron-withdrawing or electron-releasing group does not differ in activity [59].

The quinoxaline-2-carboxamide 1,4-di-*N*-oxide derivatives used as precursors for the synthesis of these new series were also evaluated against *Mycobacterium tuberculosis.* In these series, molecules substituted with an electron-withdrawing group at position 7 such as CF_3_, Cl, or F on the quinoxaline ring were the most active compounds, with the respective IC_90_ of 1.07, 1.25, and 4.65 µM. In order to explore the structural requirements for anti-TB activity, molecular modelling studies were performed on a series of 23 original quinoxaline-2-carboxamide 1,4-di-*N*-oxide derivatives, with IC_90_ values ranging from 3.86 µg to 100 µg [60]. The reliable pharmacophore generated was composed of one aromatic ring, four hydrophobic substituents, three hydrogen acceptors, and one hydrogen donor. The binding mode of these derivatives was similar to that of coumarin in novobiocin, which is known to be an inhibitor of *Mycobacterium tuberculosis* DNA gyrase active site.

#### 4.2.2. Other Structures

In order to develop new antitubercular agents, a series of 33 original quinoxaline-1,4-di-*N*-oxide derivatives variously substituted at the C2 position were synthesized and evaluated [61]. Of these, 17 showed significant activity against *Mycobacterium tuberculosis*, with the MIC ranging from 0.39 to 6.25 µg/mL and no cytotoxic effects on VERO cells (Table 11). Compounds bearing a thioether linkage and a sterically bulky aromatic group at the terminal side chain on the C2 position are the best representatives of these original series.

Additionally, the inhibitory effect of methyl, ethyl, isopropyl, and *n*-propyl esters of quinoxaline 1,4-di-*N*-oxide on *Mycobacterium tuberculosis* was assessed [62]. Of the 18 original esters of the 1,4-di-*N*-oxide synthesized, eight derivatives showed similar activity to that of isoniazid taken as reference (Table 12).

The steric effect of the ester group at position 7 plays a crucial role in enhancing biological effects. The (CH_3_)_2_CH substituent at position 7 enhances antimycobacterial activity. COOCH_3_ or COOCH_2_CH_3_ attached at position 2 on the quinoxaline ring and CF_3_ substituent at position 3 also result in increased antimycobacterial activity.

**Figure 14:**
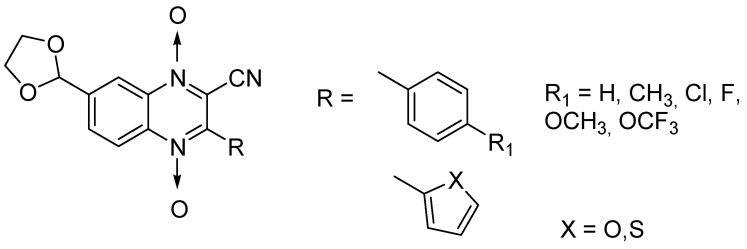
Design of the quinoxaline 1,4-di-*N*-oxide derivatives.

Influence of substitution at positions 2, 3, and 7 was also assessed in a new series of 22 new *N*-oxide-containing compounds leading to the synthesis of nine quinoxaline 1,4-di-*N*-oxide derivatives [63] (Figure 14).

For quinoxaline phenyl derivatives, MIC_90_ values ranged from 12 to 30.8 µM versus 0.1 µM for the standard drugs isoniazid and rifampicin taken as reference. Antimycobacterial activity was affected by the presence of a *para* substituent on the phenyl ring: absence of substitution led to the derivative with lowest activity while the derivatives substituted by a methoxy group displayed the highest activity. However, no clear evidence of impact from electron-withdrawing and electron-donating groups was observed. In addition, isosteric substitution of the phenyl ring by furan led to a more active compound, with MIC_90_ of 5.2 µM.

## 5. Conclusions

This review highlights the growing interest in the development of compounds bearing a quinoxaline moiety for antimycobacterial treatment, some of these compounds having reached the preclinical evaluation phase. From the published studies, both quinoxaline and quinoxaline-1,4-di-*N*-oxides with a variety of substituents in positions 2, 3, 6, and 7 showed anti-TB activity. From these data, it appears that the quinoxaline moiety represents an interesting scaffold against *Mycobacterium tuberculosis*. Some structure–activity relationships can be proposed for further investigations on this scaffold. Firstly, it appears that quinoxaline derivatives with the most potent antimycobacterial activity are unsubstituted at positions 1, 4, 5, and 8, although the presence of the pyrrolo substituent at position 1 does not result in a loss of biological activity. Substituents in positions 2 and 3 appear to play an important role in anti-TB activity as the presence of alkynyl derivatives, azide, hydrazone or acylhydrazone, chalcone, azetidinone, thiazolidinone, carboxylic acid substituent leads to significant activity. Various substituents such as thioether linkage, ester, carboxamide, chalcone, ketone, thiazolidinone, or hydrazide at position 2 lead to derivatives with potent antimycobacterial activity in quinoxaline 1,4-di-*N*-oxide series. The presence of a trifluoromethyl or methoxy group at position 3 of the quinoxaline moiety increases the activity. Substitution at positions 6 and 7 is not crucial, but derivatives substituted with ester, halogen, nitro, trifluoromethyl group, chalcone, or heterocycle at these positions exhibited excellent antimycobacterial activity. Hybridization of different pharmacophoric moieties with a quinoxaline nucleus might help to develop an effective molecule for TB treatment. These results warrant further investigations, which may allow identification of novel antitubercular candidates based on this scaffold.

## Figures and Tables

**Table 1:** Data sources and searches.

Site	Keyword 1	Boolean Operator	Keyword 2	Date	Filter
MEDLINE/PubMed (National Library of Medicine—www.ncbi.nlm.nih.gov/pubmed (accessed on 13 April 2021)	Quinoxaline	AND	Tuberculosis	01 January 2011–01 April 2021	Document type: journal articles
Web of Science (Thomson Reuters Scientific—www.webofknowledge.com (accessed on 13 April 2021)	Quinoxaline (topic)	AND	Tuberculosis (Topic)	2011–2021	Document type: only articles
Science Direct Elsevier (www.sciencedirect.com (accessed on 13 April 2021)	Quinoxaline	AND	Tuberculosis	2011–2021	Document type: only research articles

**Table 2:** Inclusion and exclusion criteria.

	Parameter	Inclusion	Exclusion
1	Language	French, English	Any other language
2	Type of study	In vitro and/or in vivo studies, biological activity	Articles that focus only on synthesis or other purely chemical parameters
3	Type of publication	Original manuscripts	Book chapters, posters, reviews
4	Search terms		Merely citing keywords in the text

**Table 3:** Chemical structure, minimal inhibitory concentration (MIC) values against *Mycobacterium tuberculosis* [30,31,32].

Compound	Structure	MIC (µg/mL)
**2**	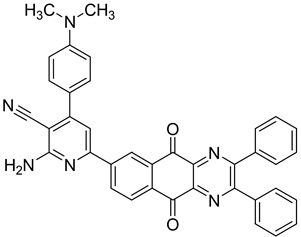	50
**3**	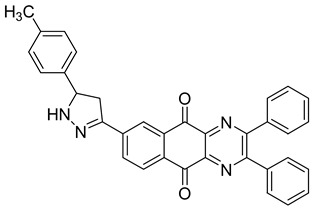	12.5
**4**	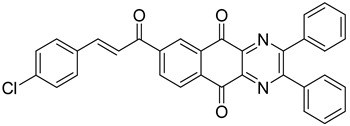	25
**5**	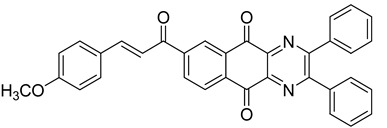	12.5
**6**	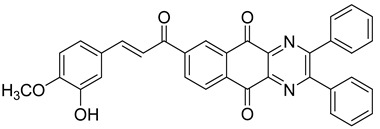	25
Rifampicin	–	0.25
Isoniazid	–	0.20

**Table 4:** Chemical structure and MIC values against *Mycobacterium tuberculosis* of the best candidates from pyrrolo[1,2-*a*]quinoxaline series.

Compound	Structure	MIC (µg/mL)
**7**	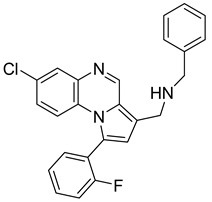	5
**8**	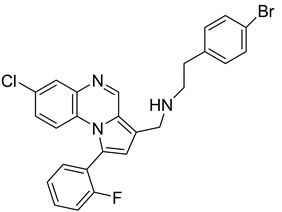	5
**9**	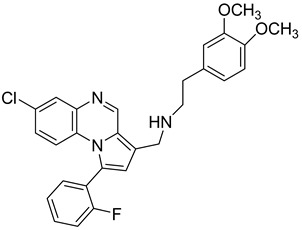	5
**10**	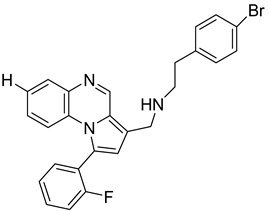	5

**Table 5:** Chemical structure of some quinoxalinyl chalcones and their MIC values against *Mycobacterium tuberculosis*.

Compound	Structure	MIC (µg/mL)
**14**	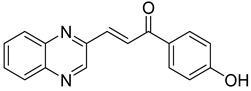	3.12
**15**	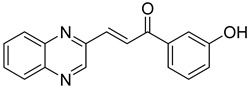	3.12
**16**	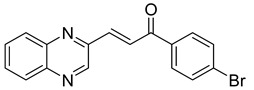	50
**17**	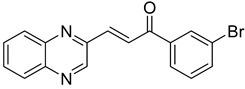	25
**18**	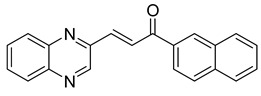	25
Streptomycin		6.25
Ciprofloxacin		3.12
Pyrazinamide		3.12

**Table 6:** MIC values against *Mycobacterium tuberculosis* of quinoxaline-derived chalcones.

Compound	Structure	MIC (µg/mL)
**19**	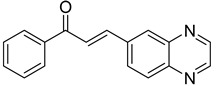	12.5
**20**	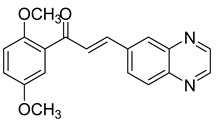	3.13
**21**	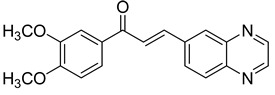	12.5
**22**	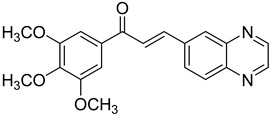	6.25
**23**	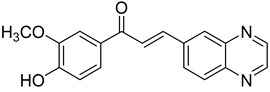	12.5
**24**	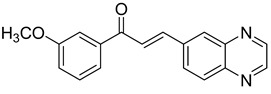	5
Rifampicin		<0.2
Moxifloxacin		<0.2
Isoniazid		0.39

**Table 7:** Chemical structure of some spiropyrrolidine tethered indenoquinoxaline heterocyclic hybrids and their MIC values against *Mycobacterium tuberculosis*.

Compound	Structure	MIC (µg/mL)
**28**	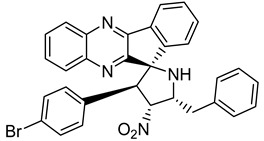	3.125
**29**	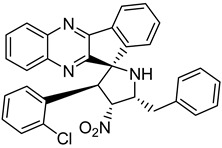	6.25
**30**	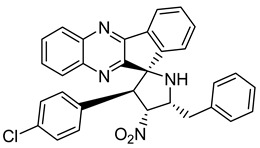	12.5
**31**	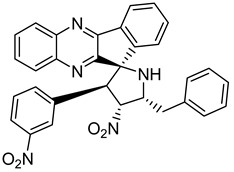	1.56
Ethambutol		1.56
Rifampicin		0.1
Isoniazid		0.05

**Table 8:** Chemical structure and MIC values against *Mycobacterium tuberculosis* of the best candidates from the quinoxaline alkynyl series.

Compound	Structure	MIC_90_ (µM)
**32**	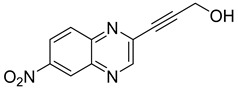	4.26
**33**	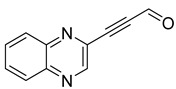	4.55
**34**	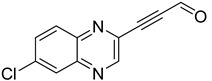	1.13
**35**	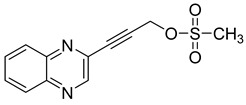	6.47
**36**	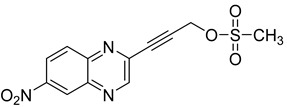	1.59
**37**	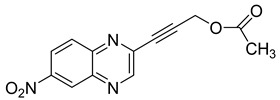	1.80
**38**	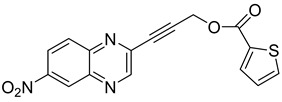	2.74
Rifampicin		0.01

**Table 9:** Antimycobacterial activity of quinoxaline derivatives based on molecular hybridization of quinoxaline 1,4-di-*N*-oxide with the chalcone and fluoroquinolone scaffolds.

Compound	Structure	MIC (µM)
**40**	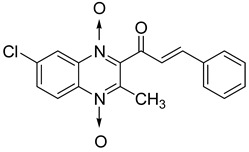	3.1
**41**	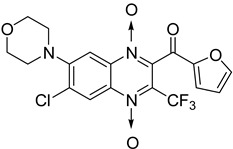	3.1
**42**	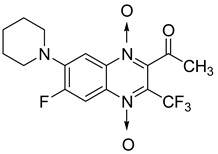	1.6
**43**	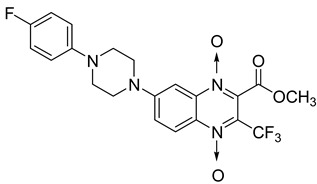	1.6
**44**	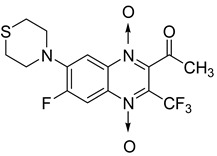	3.1
Ethambutol		6.2
Rifampicin		0.3
Isoniazid		0.04

**Table 10:** Antimycobacterial activity of quinoxaline derivatives based on molecular hybridization of quinoxaline 1,4-di-*N*-oxide with the thiazolidinone scaffold.

Compound	Structure	MIC (µg/mL)
**45**	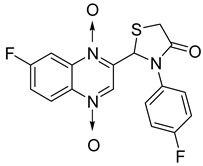	1.56
**46**	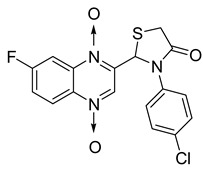	1.56
**47**	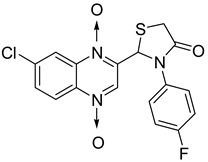	1.56
**48**	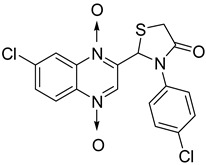	1.56
Rifampicin		0.125

**Table 11:** Chemical structure of some quinoxaline-1,4-di-*N*-oxide derivatives variously substituted at the C2 position and their MIC values against *Mycobacterium tuberculosis*.

Compound	Structure	MIC (µg/mL)
**57**	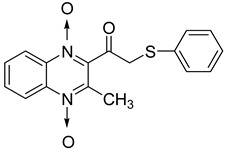	1.56
**58**	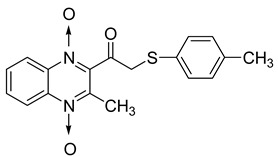	0.78
**59**	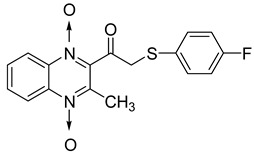	0.39
**60**	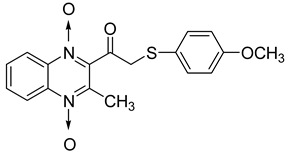	0.78
**61**	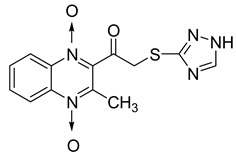	0.39
**62**	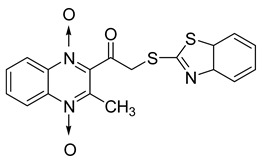	1.56
Isoniazid		0.2

**Table 12:** Chemical structure on methyl, ethyl, isopropyl, and *n*-propyl esters of quinoxaline 1,4-di-*N*-oxide series and their MIC values against *Mycobacterium tuberculosis*.

Compound	Structure	Microplate Alamar Blue Assay MIC (µg/mL)
**63**	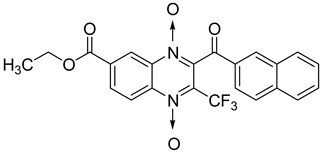	0.14
**64**	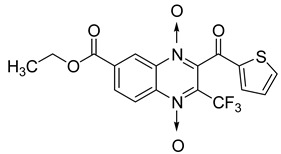	0.10
**65**	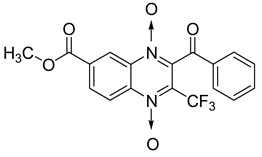	0.15
**66**	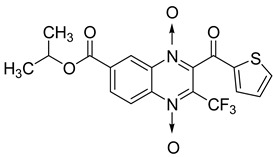	0.08
**67**	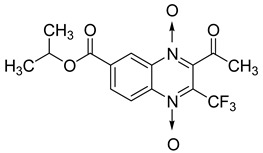	0.14
**68**	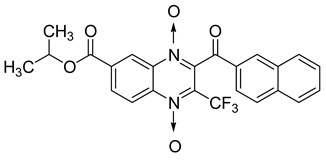	0.15
**69**	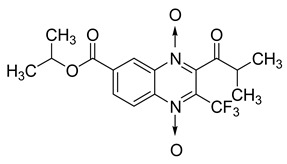	0.13
**70**	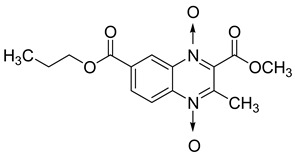	0.14
Isoniazid		0.12
